# Comparison of methods for fecal microbiome biospecimen collection

**DOI:** 10.1186/1471-2180-14-103

**Published:** 2014-04-23

**Authors:** Christine Dominianni, Jing Wu, Richard B Hayes, Jiyoung Ahn

**Affiliations:** 1 Division of Epidemiology, Department of Population Health, New York University School of Medicine, 650 First Avenue, New York, NY 10016, USA; 2 NYU Cancer Institute, 522 First Avenue, New York, NY 10016, USA

**Keywords:** Comparison, Methods, Fecal, Microbiome, Collection

## Abstract

**Background:**

Effective means are needed to efficiently collect fecal samples for microbiome analysis in large-scale epidemiological studies. Using twenty-four fecal aliquots prepared from three healthy individuals, we compared the following four fecal sample collection methods for assessment of human gut microbiome: 1) fecal occult blood test cards, held at room temperature for three days, 2) Eppendorf tubes, at room temperature for three days, 3) Eppendorf tubes with RNAlater, at room temperature, and 4) as controls, samples immediately frozen at −80°C. The 24 samples were assayed by 16S rRNA gene sequencing to compare overall microbiome structure and taxon distributions according to collection method.

**Results:**

Storing fecal occult blood test card samples at room temperature for three days did not affect total DNA purity and relative 16S rRNA bacterial gene contents, compared with fresh frozen collection. Overall microbiome structure, based on phylogenetic UniFrac index, differed significantly by subject (p = 0.001), but microbiome structure (p = 0.497) and relative abundance of major microbial taxa (phyla) (p > 0.05) did not differ significantly by collection method.

**Conclusions:**

Our findings suggest that low-cost fecal occult blood test card collection may be a feasible means of sample collection for fecal microbiome assessment in large-scale population-based studies.

## Background

The human bowel hosts trillions of gut microbial cells, the gut microbiome [1]. Although case–control investigation points to a potential role of the gut microbiome in colorectal cancer [2], large-scale prospective study of this association has been impeded by the lack of validated fecal sample collection methods suitable for large-scale studies. Our interest was in development of a fecal sample collection method that is accurate, while also being cost-efficient and easy for the study participant to use. Because fecal collections may take place outside of research clinics, we also wished to develop a fecal collection approach which would not require immediate sample processing.

To address this need, we evaluated the utility of the Beckman Coulter Hemoccult Sensa® card (Beckman Coulter, CA) for gut microbiome characterization because it offers a practical way to collect fecal samples for large-scale study. As freezing at ultralow temperatures stabilizes bacterial samples [3], we compared results for samples collected by the card method to results for samples immediately stored in Eppendorf tubes at −80°C [4]; we also included storage in Eppendorf tubes at room temperature as part of our evaluation. Finally, we were interested in evaluating the utility of collection in RNAlater, because this RNA-stabilizing agent has been shown to be suitable for samples dedicated for DNA amplification [5,6]. Our main goal was to assess the effect the different storage conditions have on gut microbiome diversity parameters including overall diversity and specific taxon abundances because different methods might differentially impact bacterial overgrowth or DNA degradation that could lead to biased assessment of these microbial parameters.

## Methods

### Study population and fecal biospecimen collection

Three healthy volunteers (2 females and 1 male) provided fecal biospecimens at NYU Langone Medical Center, New York, NY. Single fecal samples for each subject were aliquotted within 30 minutes of stool production, in duplicate using the four following collection and storage methods. In Method 1 (card) the fecal sample was smeared onto a Beckman Coulter Hemoccult Sensa® card (Beckman Coulter, CA) and kept at room temperature. In Method 2 (room temperature) fecal samples were placed in an Eppendorf tube and left at room temperature. In Method 3 (RNAlater) fecal samples were placed in an Eppendorf tube containing 1 ml RNAlater Solution® (Life technologies, NY) and left at room temperature. In Method 4 (frozen) fecal samples were frozen on collection at −80°C in a 1.5 ml Eppendorf tube. All samples were stored for three days in their respective method. We chose three days to mimic the conditions of samples being collected at home and returned to us by mail.

### Ethics statements

The study protocol was approved by the NYU Langone Medical Center Institutional Review Board. All study participants provided informed consent.

### 16S rRNA microbiome assay

After three days of storage for the four methods, genomic DNA was extracted from the 24 fecal aliquots using the PowerLyzer PowerSoil DNA Isolation Kit (Mo Bio Laboratory Inc. CA) following the manufacturer’s protocol. DNA concentration was quantified using the Synergy™ H1M microplate reader (Biotech, VM) and corresponding OD 260/280 ratio was used to check DNA purity. 16S rRNA gene amplicon libraries were generated using primers incorporating FLX Titanium adapters and a sample barcode sequence covering variable region V3 to V4 as we described elsewhere [7]. The amplicon library was sequenced using the 454 Roche FLX Titanium pyrosequencing system following the manufacturer’s instructions.

The QIIME pipeline [8] was used to process and filter multiplexed sequence reads. The UCLUST method [9] was used to cluster the filtered sequences with ≥97% similarity into Operational Taxonomic Unit (OTUs). Chimeric sequences were identified by ChimeraSlayer [10] and removed. Representative sequences from each OTU were assigned taxonomy using the Ribosomal Database Project classifier method [11] and the IMG/GG GreenGenes database of microbial genomes. A phylogenetic tree was constructed by applying the FastTree method [12] to the representative sequences.

Rarefactions of 10 to 8,414 [minimum-maximum sequence depth] randomly selected sequences from each sample were used to calculate the Shannon index, a measure of within sample diversity, and to generate rarefaction plots. Pairwise comparisons of Shannon indices by subject and storage condition were obtained by Monte Carlo permutation. All p-values were adjusted by Bonferroni correction. To measure the diversity among subjects or storage conditions, a single rarefaction was performed at a sequencing depth of 4000 so that all samples were included in analyses. Distance matrices containing all pairwise comparisons were created for unweighted (presence/absence) dissimilarity values using the UniFrac phylogenetic method [13]. Principal coordinates were computed for the unweighted distance matrices and used to generate Principal Coordinate Analysis plots (PCoA). The non-parametric method, adonis [14], was used to identify significant differences in phylogenetic distance variation by subjects and by storage condition. The Unweighted Pair Group Method with Arithmetic Mean (UPGMA) for clustering of samples was also carried out on the unweighted distance matrices [8]. A two-sample t-test was used to test for differences between the within and between group variances, with p-values adjusted by Bonferroni correction. Relative abundances of the three major phyla (Bacteroidetes, Firmicutes, Actinobacteria) were compared for the four methods, using the Mann–Whitney-Wilcoxon test, and compared by subject, using the Kruskal-Wallis test (SAS, version 9.3, SAS Institute, Cary, NC).

## Results

DNA from 24 fecal aliquots was successfully extracted and amplified. The OD 260/280 ratio, a measure of DNA purity, was greater than 1.8 in samples collected from card, room temperature, and frozen methods; DNA purity from these methods were higher than DNA purity from RNAlater (Table 1, p < 0.05). From the initial 584,367 microbial 16S rRNA sequences, 347,795 sequence reads passed filtering criteria. 16.6% of these sequences were chimeric and subsequently removed resulting in 290,110 high-quality sequence reads (12,088 ± 7,302 [mean ± SD] sequences per sample) binned into one of 5,605 OTUs. The number of sequence reads did not differ significantly according to collection methods (Table 1, p = 0.84).

**Table 1:** DNA purity and 16 s rRNA sequence reads by fecal collection method

**Method**^**a**^	**OD 260/280 (Mean ± SD)**^**b**^	**Filtered sequence reads (Mean ± SD)**^**d**^
Method 1: Card	1.86 ± 0.05	12,448 ± 8,761
Method 2: Room Temperature	1.81 ± 0.07	16,451 ± 12,004
Method 3: RNAlater	1.66 ± 0.14^c^	13,393 ± 5,909
Method 4: Frozen	1.80 ± 0.05	14,467 ± 10,030

^a^1: fecal occult blood test cards at room temperature for 3 days, 2: Eppendorf tubes at room temperature for 3 days, 3: Eppendorf tubes with RNAlater at room temperature for 3 days or 4: frozen at −80°C for 3 days.

^b^Anova was used to test for overall differences across storage methods (p < 0.005).

^c^Based on Anova results, we conducted Post Hoc TEST (LSD method) to make multiple comparisons, indicating that Method 3 resulted in lower OD 260/280 ratio (p < 0.05).

^d^Kruskal-Wallis was used to test for overall differences across storage methods (p = 0.84).

**Figure 1:**
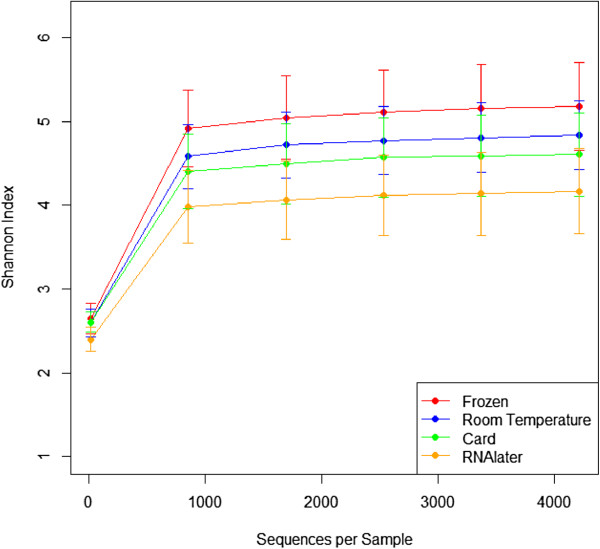
**Alpha rarefaction plot of Shannon indices (±Standard Error) according to collection method.** Card (green), Room Temperature (blue), RNAlater (orange), Frozen (red). Statistical significance was tested by using non-parametric Monte Carlo permutations (QIIME).

**Figure 2:**
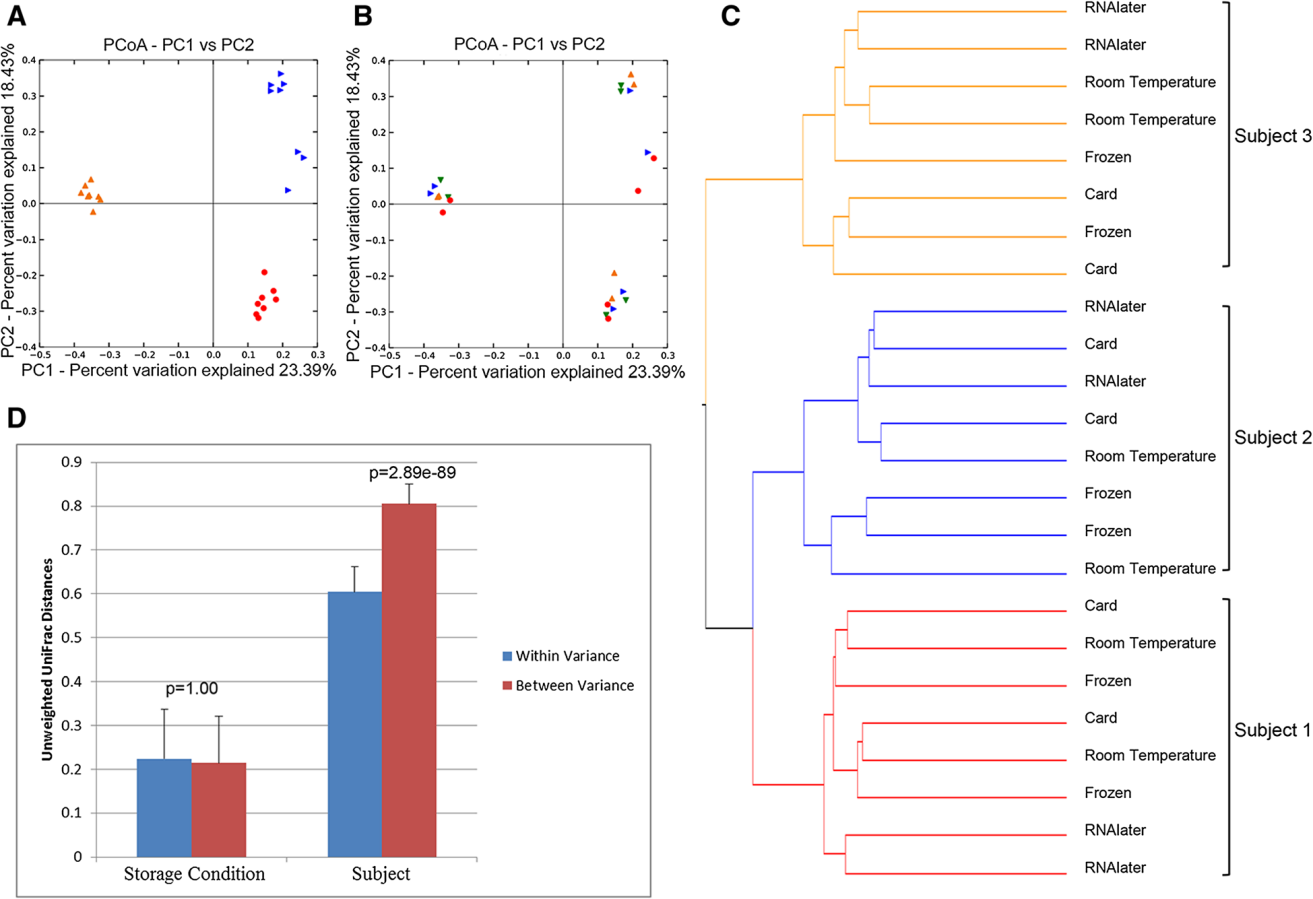
**Unweighted PCoA plots of the first two principal coordinates. A)**, **B)** The first two principal coordinates were grouped by subject (1 [red], 2 [blue], 3 [orange]) **A)** or collection method (card [green], room temperature [blue], RNAlater [orange], frozen [red]) **B)**. Adonis was used to test for significant differences in the variation in distances across subjects or collection methods using QIIME. **C)** UPGMA clustering on unweighted UniFrac distances (subject 1 [red], 2 [blue], 3 [orange]). **D)** Mean (±Std) unweighted UniFrac distances within and between sample collection methods or subjects.

Overall gut microbial diversity did not differ significantly according to the four fecal sample collection methods. The Shannon index, an indicator of gut microbial diversity, did not significantly differ by room temperature storage on either a fecal occult blood test card or in an Eppendorf tube compared to frozen samples (Figure 1, p = 0.696-1.00) but RNAlater samples tended to be less diverse than frozen samples (p = 0.072). Principal coordinate analysis based on unweighted UniFrac distances, a phylogeny-based distance metric, indicated that samples clustered by subject (Figure 2A, p = 0.001), rather than by storage condition (Figure 2B, p = 0.497). Hierarchical clustering of unweighted UniFrac distances further substantiated these findings (Figure 2C), revealing three distinct clusters by subject and not by collection method. Consistent with these findings, the gut microbial community composition varied significantly less within subjects than between subjects (Figure 2D, p = 2.89e-89). In contrast, the microbial community composition variation within collection methods was not statistically different from the variation across collection methods (p = 1.00).

**Figure 3:**
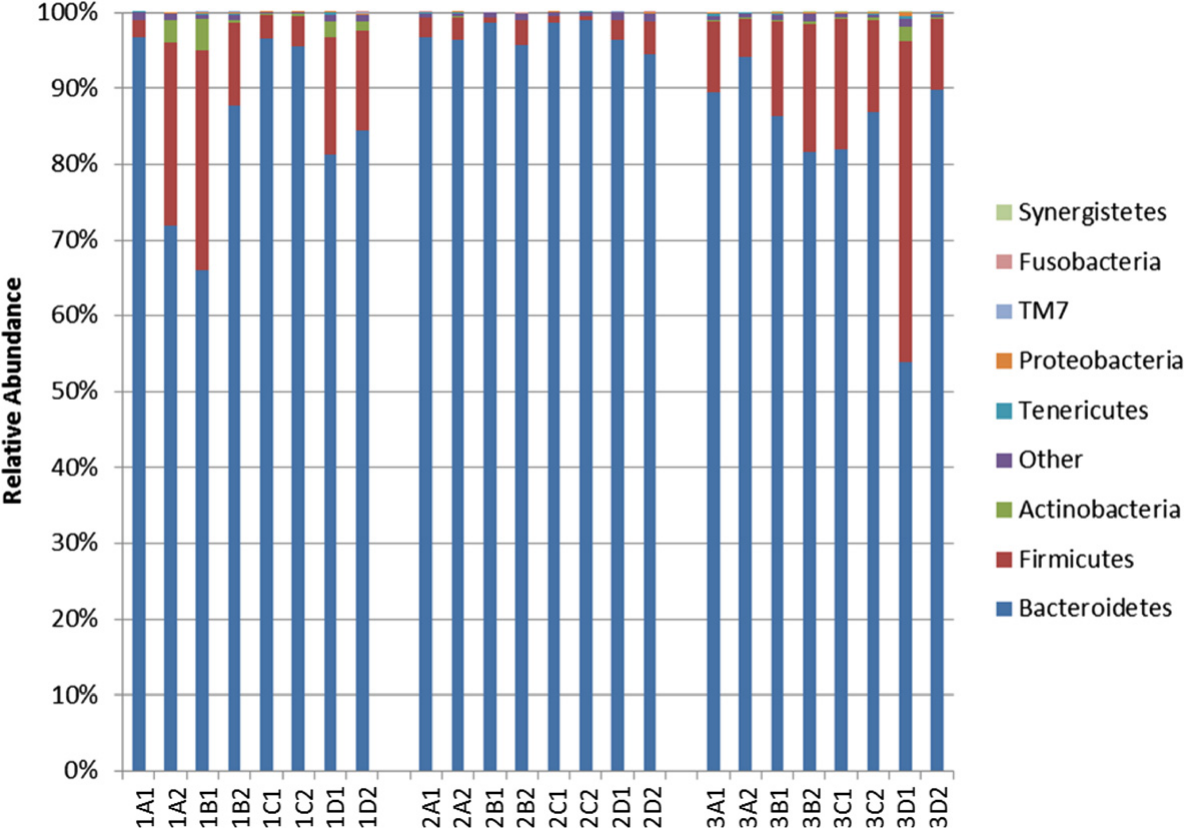
**Relative abundances of phyla by subject and by collection method.** Card (1A-3A), Room Temperature (1B-3B), RNAlater (1C-3C), Frozen (1D-3D). Kruskal-Wallis or Mann-Whitney-Wilcoxon tests were used to test for overall differences using SAS software (version 9.3).

Relative abundances of gut microbial taxa were not statistically different for any of the three test methods, when compared to relative abundances from frozen samples. The average relative abundances for the three major phyla were Firmicutes (12.2%), Bacteroidetes (86.2%), and Actinobacteria (0.7%). As shown in Figure 3, there was variability in the relative abundance of phyla by subject for Bacteroidetes (p = 0.003), Firmicutes (p = 0.0023), and Actinobacteria (p = 0.0002). For Bacteroidetes, Firmicutes, and Actinobacteria, relative abundances from samples stored in any one of the three unfrozen methods were not statistically different from relative abundances for samples immediately frozen (p > 0.05 for all).

## Discussion

We found no evidence of significant differences in gut microbial community composition and taxon distributions for storage at room temperature on a fecal occult blood test card or in an Eppendorf tube compared to immediately frozen samples. Not surprisingly, overall microbial diversity varied by subject. We found a decrease in DNA purity for samples collected with RNAlater.

Although the effect of collection container has not been previously assessed, our general observation that inter-individual differences in bacterial composition were greater than the differences by collection method is consistent with findings from previous studies. Multiple studies have tested storage durations (up to six months) and storage temperatures ranging from 20°C to −80°C; most studies [4,15,16], though not all [17,18], have found that these fecal collection methods did not significantly influence the gut microbiome diversity and taxon distribution. Two other studies reported that storage at −20°C for up to 53 days influenced specific taxa, including Bacteroidetes abundance [19] and the Firmicutes to Bacteroidetes ratio [20], however, we did not observe these trends in our study.

Samples collected with RNAlater had significantly lower DNA purity and tended to show lower microbial diversity. RNAlater is used to stabilize and protect RNA from degradation in tissue during long term storage and has been shown to also be suitable for DNA preservation [21]. However, we observed that fecal samples were very hard to disperse evenly in RNAlater during processing and that DNA purity was lower. Low-quality DNA can interfere with downstream applications including PCR amplification [22], a possible reason for the trend toward reduced Shannon indices. Two studies showed that storage in RNAlater is suitable for PCR amplification of bacterial DNA [5,6]. While the first study showed that total DNA yields from RNAlater samples were higher compared to refrigeration storage and liquid nitrogen freezing, the impact on Shannon indices was not described [5]. Using gorilla fecal samples, the second study reported that DNA purity and Shannon indices were not significantly different between RNAlater samples and samples stored at −30°C [6]. RNAlater storage increases the potential utility of stored fecal samples, so further study is warranted to determine the conditions of collection for which this reagent is suitable.

Although our study showed no differences in microbiome composition between card collection with room temperature storage and collection in Eppendorf tube with immediate freezing, we recognize that a larger series of samples may have identified some differences not found here. Also, our subjects were healthy and the collected samples may not have captured the full range of stool conditions that might be expected if subjects were ill. These considerations may be important in carrying out stool collection in different study settings.

Our findings support the use of fecal occult blood test card collections for microbiome assessment of fecal samples. These cards are commercially available and inexpensive. The small size and flat shape also makes the card easier to include in packages to be sent to participants, compared to bulkier Eppendorf tubes. Study subjects can easily collect samples on the cards. Because the cards are widely used in colorectal cancer screening [23], potential participants might also be more accepting of collecting samples in this way. A possible drawback of the Hemoccult Sensa® card is that it contains a chemical reagent used to detect blood in the stool [24] which could possibly affect gut microbiome. However, we found no evidence of a significant difference in gut microbiome in fecal samples collected by this method. Findings that results were unaffected by three-day storage at room temperature of the collection cards or Eppendorf tubes suggests that participant home-collection and mailing of these samples is suitable for epidemiological studies.

## Conclusions

Our findings suggest that fecal collection on a fecal occult blood test card or in an Eppendorf tube and storage for three days at room temperature does not substantially influence the assessment of gut microbiome. Because of the low-cost and simplicity of use, fecal occult blood test card collection may be a feasible method for large-scale population-based studies.

## Competing interests

The authors declare that they have no competing interests.

## Authors’ contributions

CD performed the statistical analysis and drafted the manuscript. JW carried out the sequencing assays and drafted the manuscript. RBH participated in the design of the study and helped to draft the manuscript. JA conceived the study, participated in its design and coordination, and helped to draft manuscript. All authors read and approved the final manuscript.
